# A Case Report of Endoscopic Resection and Laparoscopic‐assisted Retrieval of a Giant Brunner's Gland Hyperplasia

**DOI:** 10.1002/deo2.70316

**Published:** 2026-03-25

**Authors:** Kazunori Adachi, Chiharu Kawai, Miho Fujita, Hiroyuki Sakakibara, Tatsuya Kawamura, Taishi Takahara, Masahide Ebi, Naotaka Ogasawara, Makoto Sasaki, Masanao Nakamura

**Affiliations:** ^1^ Department of Gastroenterology Aichi Medical University School of Medicine Nagakute Aichi Japan; ^2^ Department of Gastroenterology Tajimi City Hospital Tajimi Gifu Japan; ^3^ Department of Pathology Aichi Medical University School of Medicine Nagakute Aichi Japan

**Keywords:** Brunner's glands, endoscopic resection, endoscopy, laparoscopy, minimally invasive surgical procedures

## Abstract

A 59‐year‐old man presented with left upper abdominal pain. Contrast‐enhanced computed tomography suggested a duodenal intussusception and a mass with mixed fat and soft‐tissue density extending from the duodenal bulb to the descending duodenum. Esophagogastroduodenoscopy identified a giant pedunculated lesion originating from the posterior wall of the duodenal bulb with a focal depressed area and marked redness. Narrow‐band imaging showed no obvious epithelial neoplastic changes. An upper gastrointestinal series demonstrated an elevated lesion approximately 5 cm in size. Based on these findings, we suspected giant duodenal Brunner's gland hyperplasia. Endoscopic resection was performed under general anesthesia due to the risk of intussusception and potential malignant transformation. En bloc resection was achieved using a scissor‐type endoscopic device with traction, and the specimen was safely retrieved under laparoscopic assistance with gentle compression. Histopathological examination revealed lobulated proliferation of Brunner's glands with fibrous septa and adipose tissue in the submucosa, confirming duodenal Brunner's gland hyperplasia.

## 1 Introduction

Brunner's gland hyperplasia (BGH) is a rare, benign duodenal lesion resulting from excessive Brunner's gland proliferation, predominantly located in the duodenal bulb. Although BGH is generally asymptomatic and often detected incidentally during endoscopy, larger lesions can cause abdominal pain, gastrointestinal bleeding, or obstruction [1, 2].

Endoscopically, BGH usually appears as a submucosal tumor‐like lesion, sometimes pedunculated with central depression, and is frequently covered by foveolar‐type epithelium. Preoperative diagnosis is challenging because conventional forceps biopsies often fail to provide definitive histological confirmation [3].

Several treatment approaches have been reported, including surgical resection, endoscopic resection, and duodenal laparoscopic and endoscopic cooperative surgery (D‐LECS) [2, 3, 4, 5, 6]. Although endoscopic resection is minimally invasive, safe specimen retrieval strategies remain underreported.

Herein, we describe a case of a giant duodenal BGH that was successfully resected endoscopically with laparoscopic assistance, emphasizing the technical considerations for safe specimen retrieval.

## 2 Case Report

A 59‐year‐old man presented with left upper abdominal pain. Computed tomography (CT) suggested duodenal intussusception, and the patient was referred to our department for further evaluation. Esophagogastroduodenoscopy (EGD) was subsequently performed.

**FIGURE 1:**
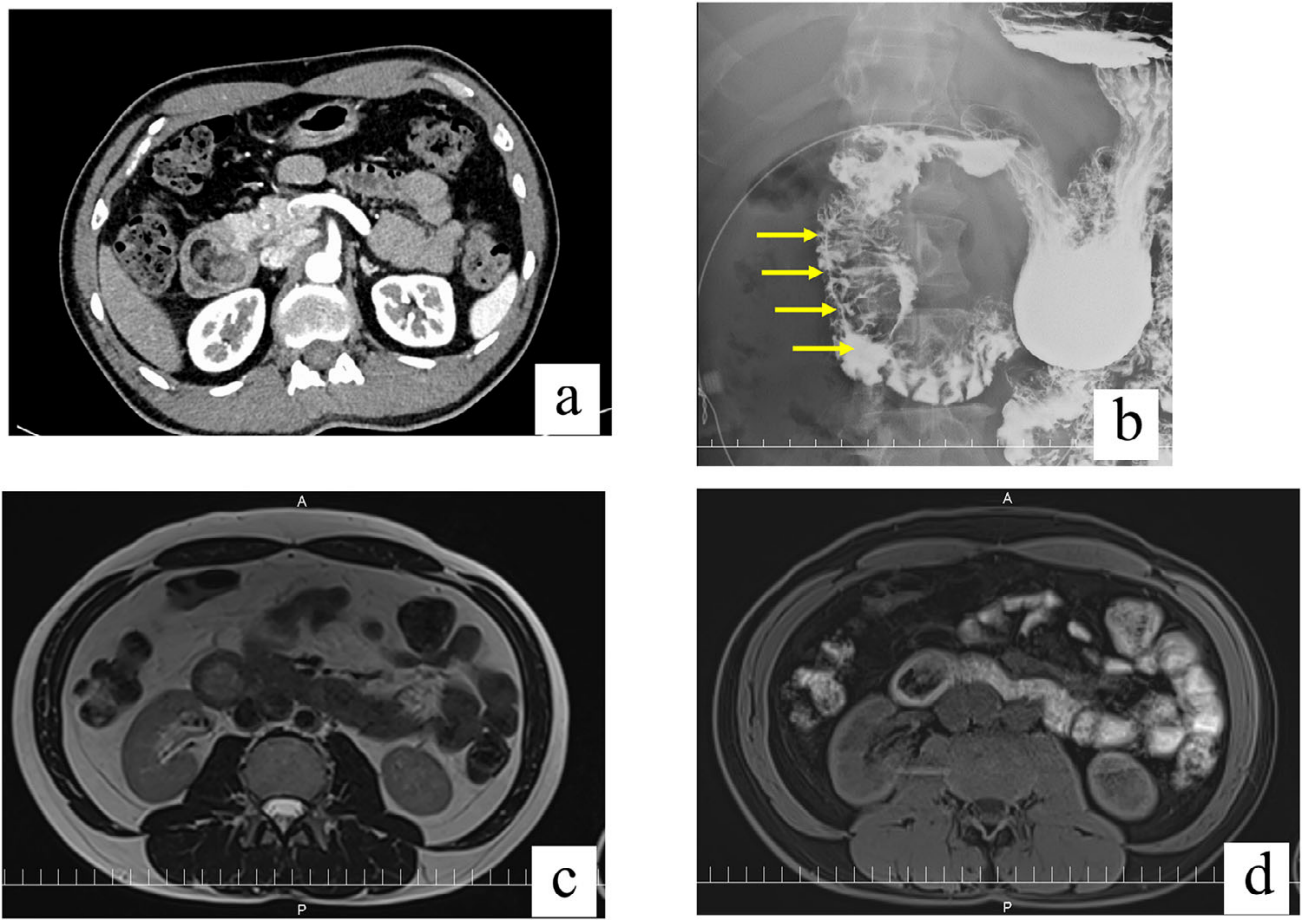
(a) Contrast‐enhanced computed tomography (CE‐CT) showing a mass lesion with mixed fat and soft‐tissue density extending from the duodenal bulb to the descending portion of the duodenum. (b) Upper gastrointestinal series showing an elevated lesion (arrow) measuring approximately 50 mm on compression views. (c) T1‐weighted magnetic resonance imaging (MRI) revealed a well‐defined mass with iso‐ to slightly high‐signal intensity. (d) T2‐weighted MRI revealed a well‐defined mass with moderately high‐signal intensity and areas of signal void.

Contrast‐enhanced CT (CE‐CT) demonstrated a mass with mixed fat and soft‐tissue density extending from the duodenal bulb to the descending duodenum, raising suspicion for intussusception (Figure 1a).

**FIGURE 2:**
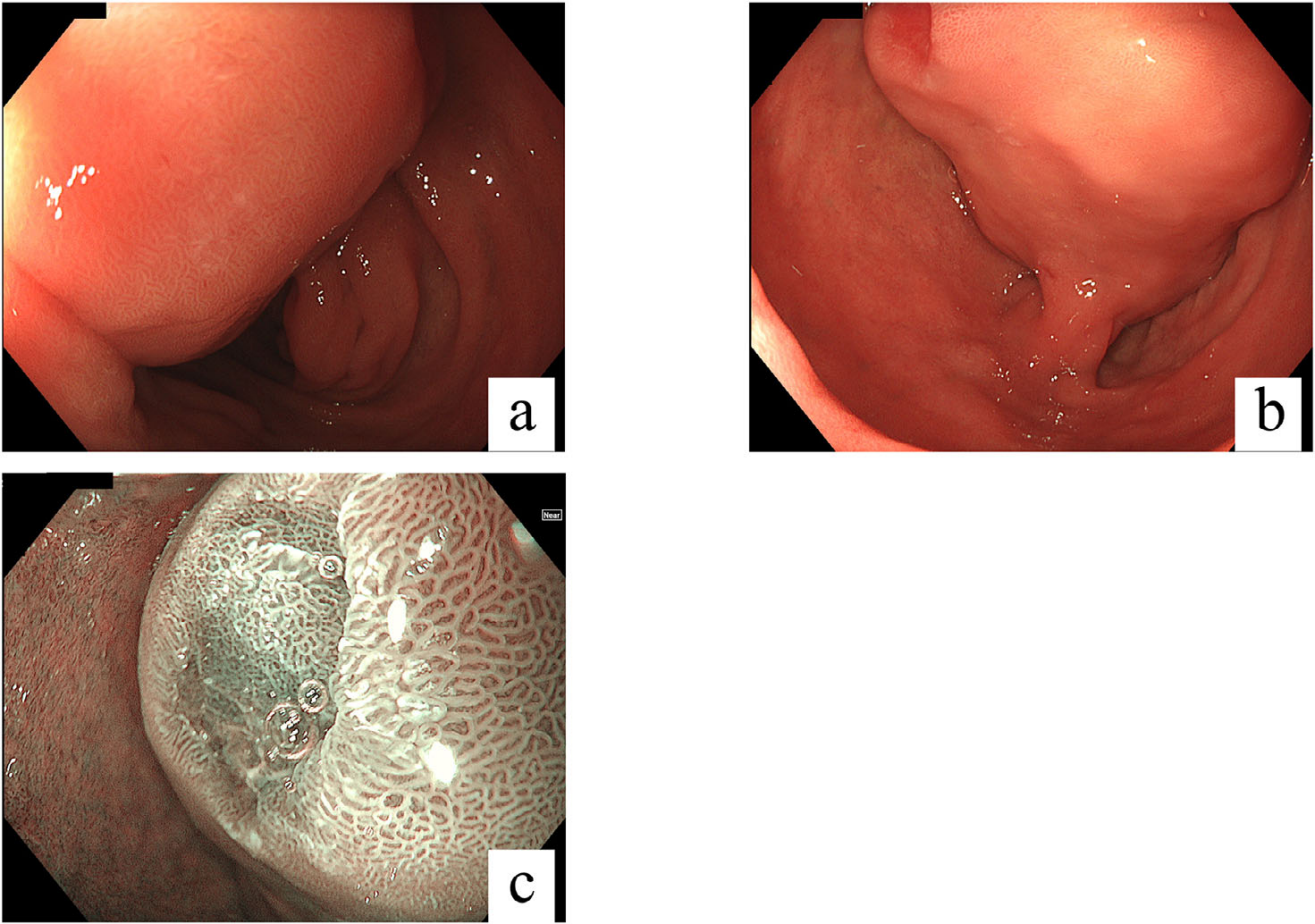
(a) A giant mass in the descending duodenum. (b) An easily mobile, submucosal tumor‐like lesion with a giant stalk arising from the posterior wall of the duodenal bulb; a focally reddened, depressed area is visible at the tip. (c) Narrow‐band imaging (NBI) showing a gastric foveolar epithelium‐like pattern on the elevated component and a similar pattern at the depressed area.

Endoscopic examination identified a large, highly mobile mass in the descending duodenum (Figure 2a). The lesion appeared as a submucosal, tumor‐like lesion with a giant stalk originating from the duodenal bulb's posterior wall. Its surface was smooth with a focally‐reddened, depressed area at the tip (Figure 2b). Narrow‐band imaging (NBI) showed a gastric foveolar epithelium‐like pattern (Figure 2c). Based on these findings, a giant duodenal BGH was suspected.

An upper gastrointestinal series confirmed an elevated lesion measuring approximately 50 mm (Figure 1b).

Magnetic resonance imaging (MRI) demonstrated a well‐defined lesion with iso‐ to slightly high‐signal intensity on T1‐weighted images and heterogeneous signal intensity on T2‐weighted images, including moderately high‐signal areas and signal voids. Although the initial abdominal pain was unlikely to be directly caused by the lesion, its size posed potential risks for future intussusception and malignant transformation. Therefore, endoscopic resection was performed. Given the large size of the lesion, risk of bleeding, and anticipated difficulty with specimen retrieval, laparoscopic ports were preemptively placed to allow for additional intervention, if needed. The procedure was performed under general anesthesia, with a 12‐mm umbilical port and bilateral 5‐mm ports adjacent to the umbilicus.

**FIGURE 3:**
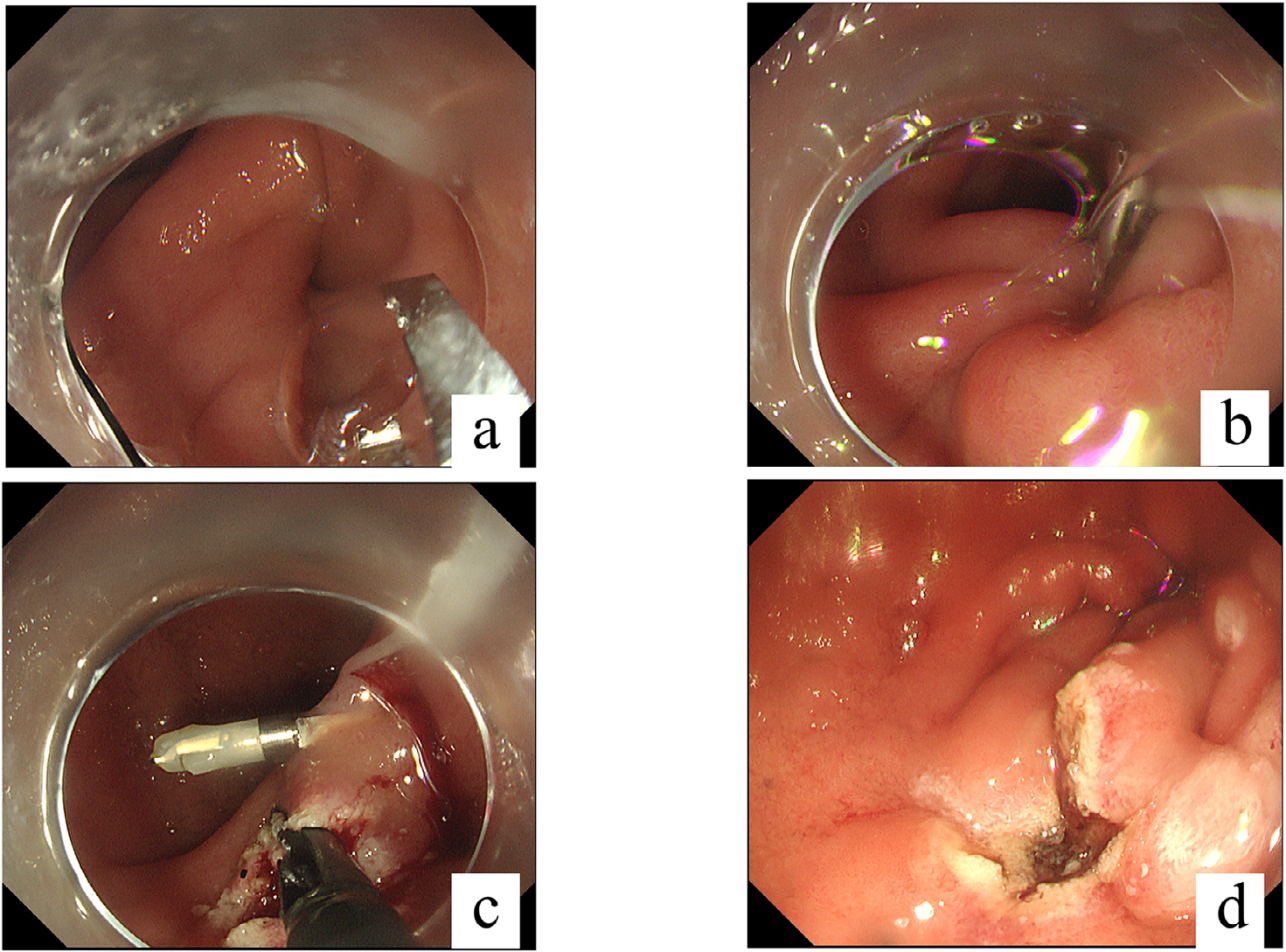
(a) The stalk arising from the posterior wall was identified, with the lesion head prolapsed into the descending duodenum. (b) Traction applied to stabilize the lesion prior to incision. (c) Lesion incision using a scissor‐type device under traction. (d) Post‐resection ulcer base.

The lesion was highly mobile and had migrated to the descending duodenum. Snare resection alone was technically challenging because of poor maneuverability. A clip with a traction thread was attached to the stalk to facilitate en bloc resection, stabilizing the lesion and pulling it back toward the duodenal bulb. En bloc resection was performed using a scissor‐type endoscopic device (Figure 3).

**FIGURE 4:**
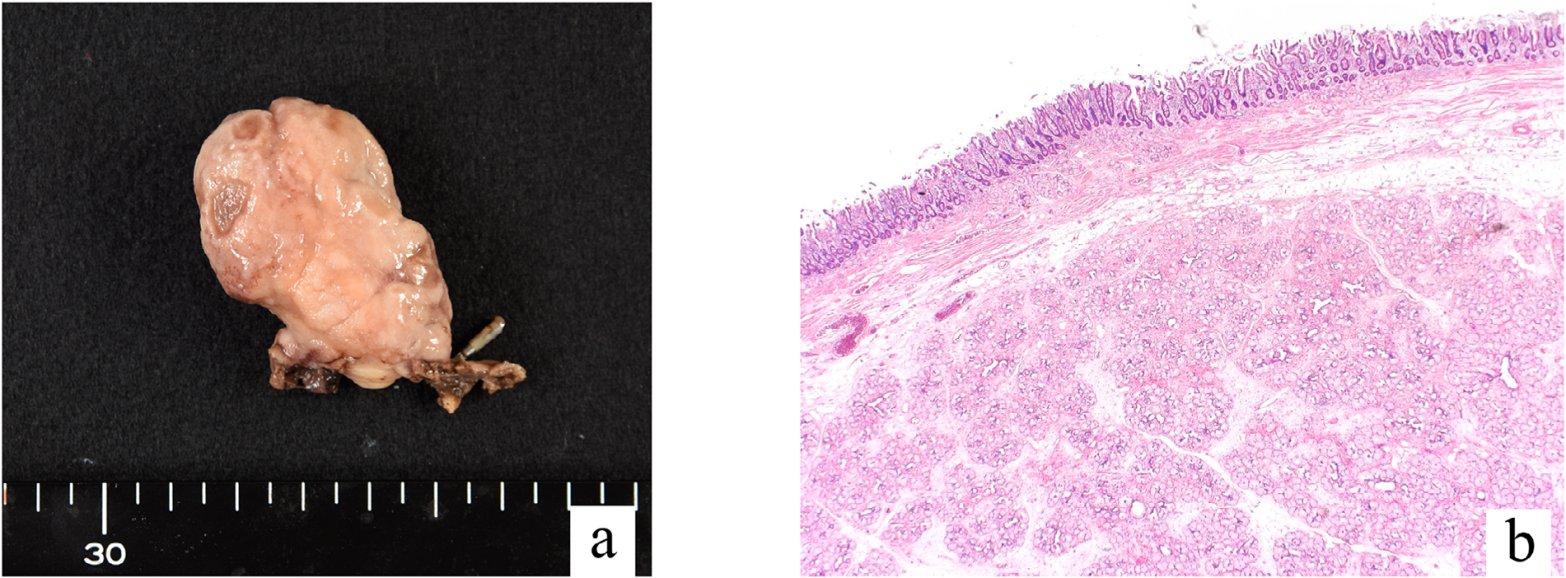
(a) Resected pedunculated lesion measuring 50 × 40 × 35 mm. (b) Histopathology (HE stain, ×40) showing non‐atypical epithelium. Covering the lesion and lobulated proliferation of Brunner's glands, accompanied by fibrous septa and adipose tissue in the submucosa.

Specimen retrieval using a retrieval net required manipulation within the duodenal bulb, where the entire lesion could not be fully visualized, and was therefore considered unsafe due to the risk of duodenal impaction. Therefore, the lesion was pulled using forceps; however, resistance was encountered when the lesion passed through the pyloric ring. Additional laparoscopic external compression of the antrum and duodenal bulb allowed partial migration of the lesion into the stomach; however, the forceps subsequently slipped off the lesion. The lesion was carefully and atraumatically grasped with a snare, and its passage into the stomach was facilitated by laparoscopic compression. A retrieval net was used for transoral retrieval, aligning the short axis of the lesion with the extraction direction, and the lesion was successfully retrieved per os (Figure S1 and Video S1). The resected specimen showed a pedunculated lesion of 50 × 40× 35 mm (Figure 4a). Histopathological examination revealed that the lesion was covered with non‐atypical epithelium. In the submucosa, a lobulated proliferation of Brunner's glands accompanied by fibrous septa and adipose tissue was observed (Figure 4b).

The final diagnosis was Brunner gland hyperplasia. No evidence of recurrence was observed during the 2‐year follow‐up period.

## 3 Discussion

BGH is a benign proliferation of Brunner's glands that presents as a submucosal tumor–like lesion and is sometimes referred to as Brunner's gland hamartoma when mixed with mature tissue components [1, 2]. BGH mainly affects individuals in their 50s–70s and is usually asymptomatic; however, large lesions may cause bleeding, obstruction, intussusception, or carcinoma [1, 2, 7]. On CT, BGH may appear as an intussusception‐like lesion showing heterogeneous density with fat attenuation; a pedunculated duodenal mass containing intralesional fat strongly suggests BGH [8]. Preoperative diagnosis is often difficult because forceps biopsy frequently yields insufficient tissue, although endoscopic ultrasound–guided fine‐needle aspiration may be helpful in selected cases [5, 6]. While BGH is generally benign, larger lesions can become symptomatic, and rare cases of dysplasia or carcinoma have been reported even in relatively small lesions [3]. Therefore, management should be individualized according to tumor size and clinical presentation.

The treatment options for BGH include surgical resection [5], endoscopic resection [5, 7], and D‐LECS [6]. In the present case, resection was performed because the lesion was a 50‐mm pedunculated tumor associated with abdominal pain and carried a potential risk of intussusception or torsion.

Traction using a clip with a thread enabled lesion stabilization, and en bloc resection was achieved using a scissor‐type device. Specimen retrieval was successfully performed with laparoscopic assistance by compressing the antrum and duodenal bulb to guide the lesion into the stomach, followed by peroral retrieval.

Previous reports of giant BGH have mainly focused on resection techniques, and discussion regarding specimen retrieval remains limited [5, 6, 7]. The short‐axis diameter has been reported to be approximately 35 mm in many cases, similar to the present case. Endoscopic full‐thickness resection for solid tumors, such as gastrointestinal stromal tumors, generally indicates that lesions ≤ 30 mm are suitable for peroral retrieval [9]. Although the lesion size in the present case slightly exceeded this threshold, the soft texture and fatty composition of BGH may facilitate peroral retrieval even for larger lesions when appropriate traction and laparoscopic‐assisted compression are used. However, alternative approaches, including surgical retrieval via duodenal or gastric incision, may be required when peroral retrieval is not feasible.

In this report, we present a retrieval strategy and discuss the importance of tumor size for pyloric passage. Although no significant intra‐abdominal adhesions were observed in this case, the feasibility of laparoscopic manipulation in patients with adhesions remains unclear, and further studies are needed to determine the size limit for pyloric passage.

When laparoscopic assistance is available in the endoscopic management of giant BGH, this approach may be useful not only for managing complications but also for cases in which specimen retrieval is difficult.

In conclusion, we report a case of giant BGH that was successfully resected en bloc using endoscopy with laparoscopic assistance. For large lesions, careful consideration of both resection and retrieval strategies is essential, and technical modifications may enable safe and minimally invasive treatment.

## Author Contributions

**Conceptualization**: Kazunori Adachi, Naotaka Ogasawara, and Makoto Sasaki. **Investigation**: Kazunori Adachi, Chiharu Kawai, Hiroyuki Sakakibara, and Miho Fujita. **Endoscopic procedure**: Kazunori Adachi, Chiharu Kawai, and Masahide Ebi. **Pathological diagnosis**: Taishi Takahara. **Data curation (image and video editing)**: Chiharu Kawai and Tatsuya Kawamura. **Writing – original draft**: Kazunori Adachi. **Final approval of the manuscript**: Masanao Nakamura, All authors have read and approved the final manuscript.

## Funding

The authors have nothing to report.

## Ethics Statement

All procedures involving human participants were conducted in accordance with the ethical standards of the institutional and/or national research committee and with the Declaration of Helsinki and its later amendments.

## Consent

Written informed consent for publication of this case report was obtained from the patient.

## Conflicts of Interest

The authors declare no conflicts of interest.

## Supporting information




**FIGURE S1**: Laparoscopic external compression applied from the duodenal bulb (arrow) toward the gastric antrum to facilitate specimen migration.

**VIDEO S1**: Endoscopic retrieval of the resected lesion. The lesion was initially pulled into the stomach using forceps, but became lodged at the pyloric ring. Additional laparoscopic external compression of the antrum and duodenal bulb was attempted but was unsuccessful. The lesion was then carefully and atraumatically grasped with a snare, and combined laparoscopic compression enabled successful migration into the stomach.
